# NHC stabilized copper nanoparticles *via* reduction of a copper NHC complex†

**DOI:** 10.1039/d3cc02745g

**Published:** 2023-07-11

**Authors:** Robert Richstein, Constantin Eisen, Lingcong Ge, Monnaya Chalermnon, Florian Mayer, Bernhard K. Keppler, Jia Min Chin, Michael R. Reithofer

**Affiliations:** a Institute of Inorganic Chemistry, Faculty of Chemistry, University of Vienna Währinger Straße 42 Vienna 1090 Austria michael.reithofer@univie.ac.at; b Institute of Materials Chemistry and Research, Faculty of Chemistry, University of Vienna Währinger Straße 42 Vienna 1090 Austria; c Institute of Inorganic Chemistry – Functional Materials, University of Vienna Währinger Straße 42 Vienna 1090 Austria jiamin.chin@univie.ac.at

## Abstract

The bottom-up synthesis of plasmonic NHC@CuNPs from common starting reagents, *via* the formation of the synthetically accessible NHC–Cu(i)–Br complex and its reduction by NH_3_·BH_3_ is reported. The resulting NHC@CuNPs have been characterized in detail by XPS, TEM and NMR spectroscopy. The stability of NHC@CuNPs was investigated under both inert and ambient conditions using UV-Vis analysis. While the NHC@CuNPs are stable under inert conditions for an extended period of time, the NPs oxidize under air to form Cu_*x*_O with concomitant release of the stabilizing NHC ligand.

## Abstract 2

NHC@CuNPs are bottom-up synthesized using NHCCuX as a precursor. Detailed NHC-binding and stability studies of the NHC@CuNPs show that they behave differently to typical NHC@Cu surface, which is important for development of new CuNP catalysts.

## Graphical abstract

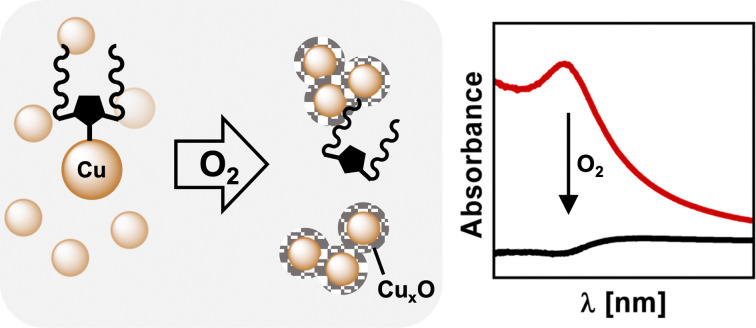

Soon after the first N-heterocyclic carbenes (NHCs) emerged as persistent ligands in organometallic catalysis, NHCs were found to also be excellent surface ligands for metallic surfaces and nanoparticles (NPs).^1^ Due to their strong σ-donation and structural versatility,^2^ NHCs are an attractive alternative to commonly used phosphor- or sulfur-based stabilizing ligands to provide the requisite robustness to metal surfaces and NPs when applied in challenging conditions. Based on these stability improvements, a variety of NHC-stabilized precious metal-containing nanoparticles (NHC@NP) have been synthesized.^1*d*,3^ Examples range from NHC@AuNPs with high colloidal stability for potential biomedical applications^4^ to NHC@NPs with high catalytic activity,^5^ including platinum,^6^ palladium^7^ and ruthenium NPs.^8^ However, literature reports on earth-abundant metal-containing NHC@NP such as NHC@CuNPs remain scarce.^9^ Despite the limited reports on NHC@CuNPs,^9*c*,*e*,10^ the interactions of NHC with Cu and surface-bond Cu(i) oxide species (Cu_*x*_O) have been thoroughly investigated.^11^ Based on these reports, NHCs bind firmly to Cu/Cu_*x*_O surfaces and mitigate oxidation or help with the etching of Cu_*x*_O from the oxidized Cu surfaces.^11*a*,*d*^ Further, binding characteristics of NHC on Cu/Cu_*x*_O surfaces have been calculated by various DFT models and verified by low-temperature scanning tunneling microscopy (LT-STM) and X-ray photoelectron spectroscopy (XPS).^9*g*,10*a*,11*b*,*c*,*e*,*f*^ Despite these solid fundamentals, the synthesis of unsupported NHC@CuNPs in dispersion has received limited attention, with only one example of colloidal NHC@CuNPs having been reported thus far, to the best of our knowledge.^9*e*^ Using NHC–borane adducts (NHC–BH_3_) and mesityl copper as starting materials (Scheme 1, left) Frogeneux *et al.* synthesized the first NHC@CuNPs in a bottom-up approach, whereby heating of NHC-BH_3_ during the reduction process, releases the necessary borane species for reducing the Cu(i) source and allowing the *in situ* attachment of the NHC ligand on the surface of obtained Cu(0) nanoparticles.^9*e*^ Herein, we report an alternative synthesis of CuNPs stabilised by a dodecyl-NHC ligand (Scheme 1, right). The developed synthesis route follows a bottom-up NP preparation using only common reagents including copper oxide (Cu_2_O), the corresponding imidazolium salt 1 and a mild borane-reducing agent in order to obtain NHC@CuNPs. Successful anchoring of NHCs on the CuNP surface was thoroughly investigated by XPS and additionally supported by multinuclear NMR spectroscopy. Furthermore, stability data of NHC@CuNPs under inert and ambient conditions was determined by UV-Vis and NMR as well as XPS, providing insights into the interplay of NHC ligand and CuNP after an oxidation event.

**Scheme 1:**
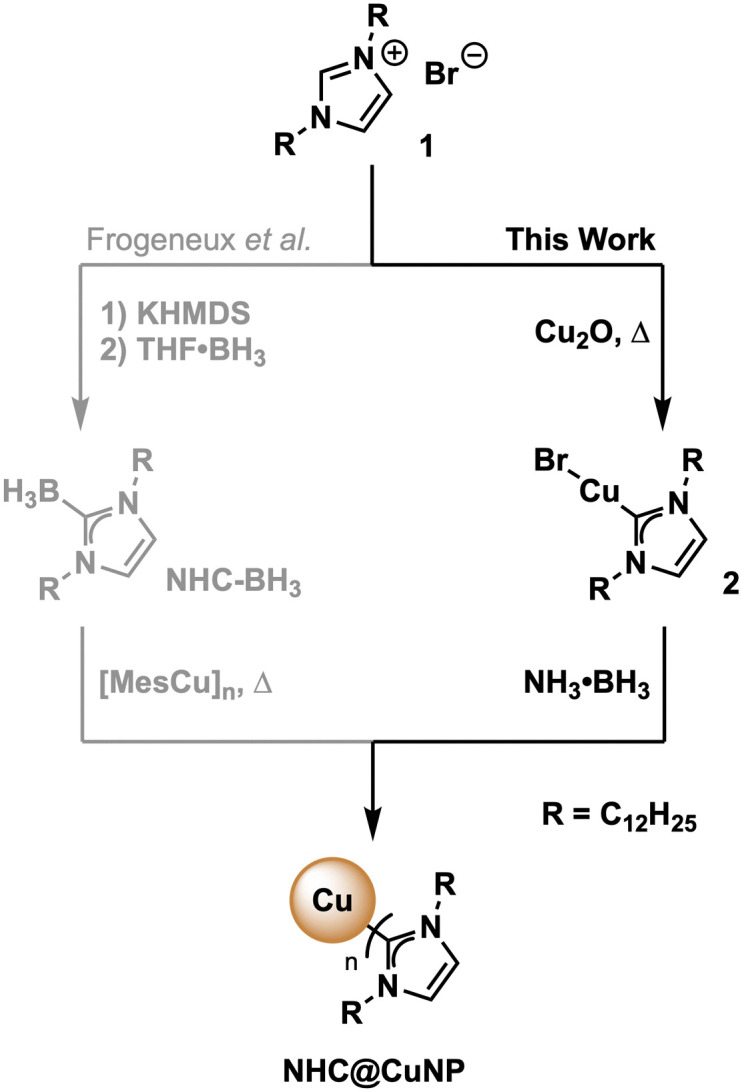
Synthesis of NHC@CuNPs. Left: Method by Frogeneux *et al. via* NHC–BH_3_ and thermal decomposition in the presence of mesityl copper ([MesCu]_*n*_); right: method presented in this work *via* NHC–Cu(i) complex 2 followed by controlled reduction with ammonia borane (NH_3_·BH_3_) in THF.

In order to obtain the final NHC@CuNPs, first the precursor NHC–Cu(i) complex 2 was synthesized based on a procedure adapted from Lu *et al.*^9*d*,12^ In short, the corresponding imidazolium salt 1 and Cu_2_O were heated under inert conditions yielding 2 with sufficient purity upon filtration. 2 was then used in a common bottom-up NHC@NP synthesis procedure, where complex 2 is reduced in a controlled manner by the addition of a borane-based reducing agent in a specific molar ratio. In this study, sodium borohydride (NaBH_4_) as a strong and ammonia borane (NH_3_·BH_3_) as a weaker reducing agent were evaluated in the formation of NHC@CuNPs. Using 2, 1, or 0.5 equivalents of NaBH_4_ as reducing agent showed that NaBH_4_ is too strong to generate colloidally stable NHC@CuNPs; even at low molar ratios of NaBH_4_ no colloidal NHC@CuNPs was obtained. Rather, the use of NaBH_4_ under all tested conditions led to the formation of bigger Cu(0) aggregates which precipitated immediately after the addition of NaBH_4_. On the other hand, NHC@CuNPs synthesised using NH_3_·BH_3_ as reducing agent showed good stability across the tested reducing agent with an observed optimum of 1 eq. of NH_3_·BH_3_ over a reaction time of 24 h (see ESI† for details). NHC@CuNPs generated *via* the optimized parameters were purified by 5 cycles of centrifugation and redispersion in toluene. The obtained NHC@CuNP pellet was finally dried *in vacuo* yielding a black solid readily re-dispersible in toluene. The dark red dispersion of NHC@CuNPs in toluene prepared by this procedure shows particles with an average diameter (d) of ∼8 nm (see TEM, Fig. 2D) and a strong absorption at 584 nm indicating the plasmonic character of these NPs. To verify the binding of the NHC ligand to the copper surface, we first employed ^1^H, ^13^C and multinuclear NMR spectroscopy.^13^ Recorded NMR spectra confirm the successful NHC formation by the absence of imidazolium salt-related signals (C^2^–H, Fig. 1) and the appearance of the characteristic carbene (C^2^) in complex 2 and NHC@CuNPs with ^13^C-NMR signals at 177.1 (2) and 176.9 ppm (NHC@CuNP), respectively (Fig. 1). Interestingly, the C^2^ signal of NHC coordinated on CuNP surface is slightly higher field-shifted (*Δ* = 0.2 ppm) compared to 2, similar to NMR data of silica-supported IMes@CuNP reported by Kaeffer *et al.*^10*a*^ Although the chemical shift difference between complex and nanoparticle typically provides a good indication for successful NHC binding to the NP surface, the effects observed in NHC@CuNPs are too small to be conclusive. As such, the binding of the NHC ligand to the CuNP was further investigated through XPS analysis. The corresponding N 1s spectra of NHCs shows two peaks, while the free imidazolium salt 1 shows a main signal at 401.3 eV (Fig. 2A and ESI,† Fig. S2). Observed peaks for NHC@CuNPs at 400.7 and 398.5 eV can be assigned to the NHC structure with a N–C^2^ and quaternary N=C contribution,^11*d*^ respectively, and show no unbound imidazolium species as previously reported by Frogeneux *et al.*^9*e*,14^ Furthermore, the oxidation state of copper is investigated by XPS. In the Cu 2p high resolution spectra, besides a Cu(0)/Cu(i) peak, a small amount of Cu_*x*_O was detected (Fig. 2B).^15^ We attribute the formation of Cu_*x*_O to the experimental setup, where the NHC@CuNPs have to be handled in air for a short amount of time. However, in comparison to the Cu(0)/Cu(i) peak the formation of Cu(ii) is not observed as previously reported for NHC modified Cu surfaces.^11*d*^ As the signals of Cu(i) and Cu(0) at ∼932.5 eV are hard to distinguish§§According to Biesinger: XPS of Cu(0) of bulk metal results in a peak at 932.6 eV, while Cu(i) in Cu_2_O has a contribution at 932.2 eV.^15^ Auger electron spectra in the region of 510–560 eV were recorded (Fig. 2C).^15^ In Auger spectroscopy, the energy difference between a core orbital and an electron in an outer orbital is measured and the spectra have a finer differentiation between the chemical state of an atom.^16^ In this region Cu(0) (567.9 eV) and Cu(i) (Cu_2_O: 570.4 eV) show separated peaks with a characteristic peak shape.^17^ In order to evaluate whether the Cu(i) signal arises from air oxidation, Cu_2_O powder as reference material was measured. When comparing the binding energy of pure Cu_2_O powder with the binding energy of our CuNPs samples, no Cu(i) was observed, indicating no surface oxidation of NHC@CuNPs when kept under a strict inert atmosphere. As the reference copper foil could not be kept under an inert atmosphere, the analysis also shows a significant signal arising from Cu(i) (569.8 eV). The herein synthesised nanoparticles show a single peak associated with Cu(0) at 567.8 eV, confirming the predominant elementary state of the nanoparticles when compared to the reference materials. In order to test the stability of the NHC@CuNPs towards air, suspensions of NHC@CuNPs were kept open in air. Upon contact with air, the dispersion immediately discolored and subsequently a black non-redispersible precipitate was formed. The visible decomposition was quantified by UV-Vis spectroscopy and the decomposition products were investigated by multinuclear NMR spectroscopy and XPS analysis. When NHC@CuNP suspensions are kept under argon, the nanoparticle suspensions exhibit a plasmonic resonance at 584 nm for up to 48 h (see ESI,† Fig. S4). This characteristic peak vanishes within seconds when the argon atmosphere in the sample cuvette is replaced with air, as the cuvettes are not air-tight (Fig. 2F). The vanishing UV-Vis peak indicates the formation of oxide species on the particle surfaces as only metallic copper nanoparticles show a plasmonic resonance. To further investigate whether the NHC@CuNPs indeed underwent oxidation, Cu 2p XPS spectra of the black precipitate were recorded (see ESI,† Fig. S1). After oxidation the spectrum shows significant peak broadening, which is characteristic for Cu_*x*_O. Deconvolution reveals a broad signal at 946.1 eV associated with Cu(i) satellite species. Furthermore, the Cu(0/i) peak splits into two contributions with a significant Cu(i) contribution at 931.3 eV indicating the formation of Cu(i) species during oxidation. However, the XPS spectra still show the presence of elemental copper, which can be explained through the formation of a passivated Cu_*x*_O shell with a bare copper core.^11*a*,11*d*^ TEM micrographs of the black precipitate show similarly sized and shaped particles (*d* ∼ 8 nm) aggregated in bigger clusters, explaining the non-re-dispersibility of the particles after contact with air. The clustering of oxidized NPs is associated with the full or partial loss of steric shielding provided by the NHC ligand (Fig. 2E). To further investigate the decomposition products multinuclear NMR spectroscopy was applied. In the ^1^H,^13^C-HMBC NMR spectrum the ^13^C signal of the NHCs C^2^ at 176.6 ppm disappeared while in the ^1^H spectrum the C^2^–H imidazolium signal at 7.20 ppm reappeared (Fig. 3). The presence of an imidazolium signal indicates the dissociation of the NHC ligand from the copper surface. The ligand undergoes rapid protonation in the process, forming the corresponding imidazolium species 1. Changes in the chemical environment of the NHCs were also detected by XPS (see ESI,† Fig. S1 and S2) analysis. In the N 1s spectrum, contributions at 401.4 (N–C^2^) and 399.1 eV (N=C) were observed which are attributed to the formation of imidazolium 1 liberated from the copper surface upon oxidation. Furthermore, the obtained supernatant was investigated by high-resolution mass spectrometry (HR-MS, see ESI,† Fig. S17) revealing the presence of imidazolium 1 as the main compound. The combined results of NMR spectroscopy, XPS analysis, and HR-MS reveal the interplay of the CuNP and the NHC ligands during an oxidation event. Upon air exposure, Cu_*x*_O species form on the surface of CuNPs. Subsequently, the NHC–CuNP bond is broken and free NHCs are protonated and converted by ambient humidity into the respective imidazolium 1. Comparing these results to the work of Crudden and coworkers using NHCs on Cu/Cu_*x*_O surfaces, no other organic or organometallic species related to the interaction of NHC with formed Cu_*x*_O were identified.^11*a*^ Furthermore, XPS and NMR data suggest no formation of stabilizing NHC–Cu_*x*_O bonds on the oxidized CuNPs surface.^11*c*^ Since imidazolium 1, the predominant species present after an oxidation event, allows only weak interaction with the oxidized surface of such CuNPs, NPs aggregate into clusters (Fig. 2E) and precipitate from the dispersion.

**Fig. 1:**
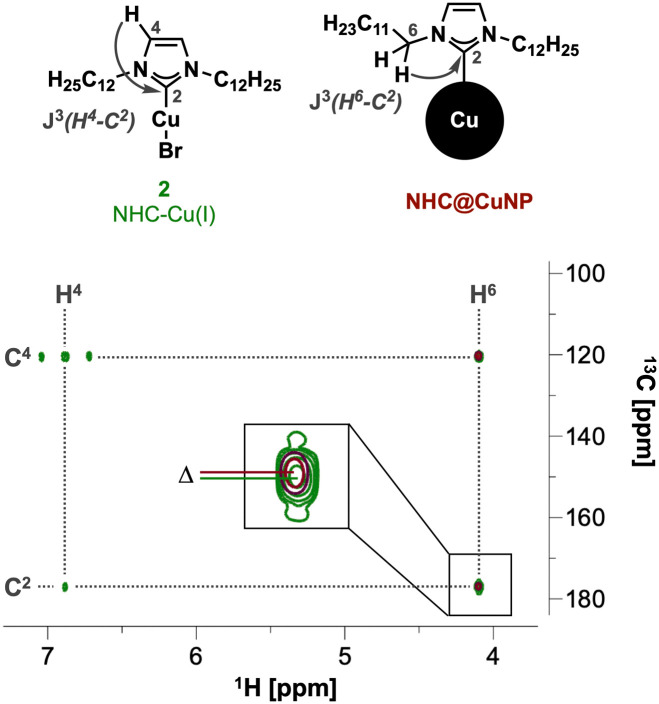
Comparison of ^1^H/^13^C-HMBC-NMR spectra of the NHC@CuNP (red) and NHC complex 2 (green). Detailed look on the C^2^ peak reveals *Δ* = 0.2 ppm going from complex to NP coordination.

**Fig. 2:**
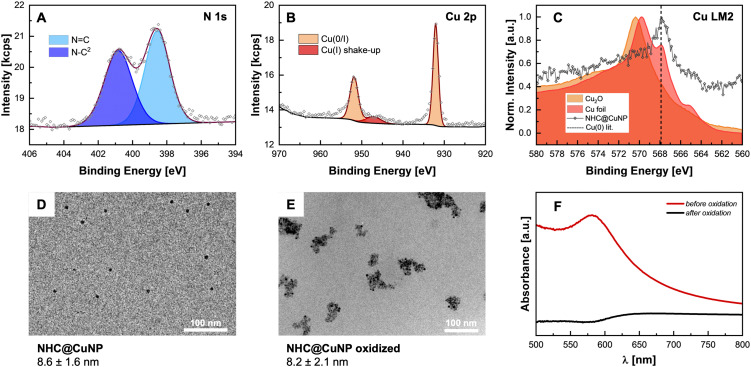
(A and B) High resolution N 1s and Cu 2p XPS scans of NHC@CuNPs; (C) Cu LM2 Auger spectra of NHC@CuNPs and reference materials; (D) and (E) TEM micrographs of NHC@CuNPs before and after oxidation, respectively: (F) UV-Vis spectra of NHC@CuNPs before and after oxidation.

**Fig. 3:**
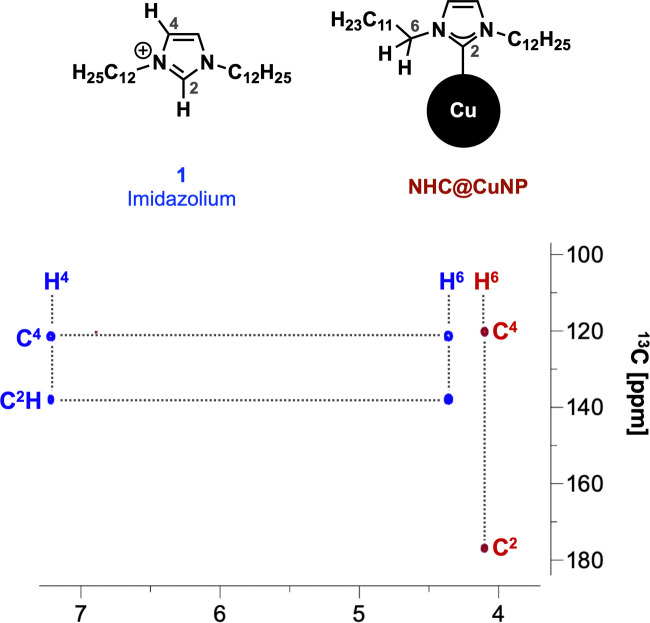
Comparison of ^1^H/^13^C-HMBC NMR spectra of NHC@CuNPs before (red) and after oxidation showing the presence of imidazolium 1 after oxidation (blue).

In conclusion, we report a straightforward two-step protocol for the synthesis of NHC@CuNPs *via* the NHC–Cu(i) complex 2 and subsequent reduction using easily accessible reagents. Obtained NHC@CuNPs were fully characterized, and their stability was assessed by monitoring their plasmonic character in inert and ambient conditions. Anchoring of NHCs on the CuNP surface did not notably suppress the oxidation of CuNPs but allowed a detailed insight into the interplay of the Cu surface and the NHC ligand during exposure to air. Comprehensive characterization of CuNPs after oxidation revealed significant differences compared to Cu surface bound NHCs studied by others. Oxidation of NHC@CuNPs generate CuNPs passivated with a Cu_*x*_O layer and simultaneous leaching of NHCs from the Cu_*x*_O layer is observed, resulting in rapid clustering of oxidized CuNPs. This work bundles synthesis, characterization, and stability investigations of NHC@CuNPs for the first time and assembles a foundation for further improvements of oxidation-resilient CuNPs stabilized by NHCs ready for catalytic applications.^18^

M. R. R. and J. C. thank the University of Vienna for financial support. C. E. thanks the Mahlke-Obermann Stiftung for the provision of a PhD Scholarship. All authors thank the NMR Centre, MS Centre and the Core Facility “Interface Characterization”, Faculty of Chemistry, University of Vienna. This project was supported by the Austrian Science Fund (FWF) stand-alone grant no. P-34662 (M. R. R.) and by ERC CoG no. 101002176 (J. C.). This research was supported by the Scientific Service Units of IST Austria through resources provided by Electron Microscopy Facility.

## Conflicts of interest

There are no conflicts to declare.

## Supplementary Material

CC-059-D3CC02745G-s001
