# Supplementary material for: NHC stabilized copper nanoparticles via reduction of a copper NHC complex
Source: Chem Commun (Camb). 2023 Jul 11;59(64):9738–41. doi: 10.1039/d3cc02745g (PMC10408246; doi:10.1039/d3cc02745g)
Supplement: CC-059-D3CC02745G-s001 [file CC-059-D3CC02745G-s001.pdf]

Electronic Supplementary Material (ESI) for ChemComm.
This journal is © The Royal Society of Chemistry 2023

Electronic Supporting Information

NHC-stabilized Copper Nanoparticles *via* Reduction of a Copper NHC Complex

*Robert Richstein[a#], Constantin Eisen[a#], Lingcong Ge[a], Monnaya Chalermnon[a], Florian Mayer[b], Bernhard K. Keppler[a], Jia Min Chin[c]*and Michael R. Reithofer[a]**

[a] Institute of Inorganic Chemistry, Faculty of Chemistry, University of Vienna, Währinger Straße 42, 1090 Vienna, Austria. Email: michael.reithofer@univie.ac.at

[b] Institute of Material Chemistry and Research, Faculty of Chemistry, University of Vienna, Währinger Straße 42, 1090 Vienna, Austria

[c] Institute of Inorganic Chemistry – Functional Materials, University of Vienna, Währinger Straße 42, 1090 Vienna, Austria. Email: jiamin.chin@univie.ac.at

[#] *These Authors contributed equally.*

Materials and Methods **2**
Synthesis of NHC Complex 2 .... **3**
Synthesis of NHC@CuNPs **3**
High Resolution XPS Spectra **4**
XPS Survey Spectra of NHC@CuNPs **5**
UV-Vis Stability Study under Argon Atmosphere **5**
TEM Micrograph of NHC@CuNPs **6**
^{13}C- and ^{1}H-NMR Spectra **7**
Imidazolium salt 1 7
NHC Complex 2 8
NHC@CuNPs 9
NHC@CuNPs after air exposure 11
High Resolution Mass Spectrometry of NHC@CuNP after Oxidation **12**
References **13**

Materials and Methods

All experiments, if not stated otherwise, were performed under argon atmosphere in a MBraun Unilab Pro glovebox. Commercially available reagents were used without further purification. Dry solvents (toluene and THF) were obtained from Acros/Fischer or Sigma Aldrich and stored over activated molecular sieves (3 Å) inside a glovebox. $CDCl_3$ (99,80% D, <0.01% H_2O) was obtained from Euroisotop and dried over $CaCl_2$, redistilled and stabilized with silver wire. Thin layer chromatography was performed using silica 60 F254 plates purchased from EDM Millipore. All molecular products were fully characterized using multinuclear NMR spectroscopy and high-resolution mass spectrometry. Obtained nanomaterials were characterized using XPS, UV-Vis and NMR multinuclear spectroscopies as well as TEM.

Imidazolium salt **2** was synthesized following a procedure from literature.[1]

X-ray photoelectron spectroscopy (XPS) was performed on a Nexsa Photoelectron Spectrometer (Thermo Fisher Scientific, UK) provide by the Core Facility "Interface Characterization", Faculty of Chemistry, University of Vienna. Samples were drop casted from suspensions in toluene onto freshly cleaned silicon wafer and dried for 48 h in an inert atmosphere at room temperature. The silicon wafers (~0.25 cm^2) were cleaned by sonication in methanol and acetone with subsequent drying under reduced pressure. Element specific high-resolution spectra for Carbon (C 1s 279-298 eV), Nitrogen (N 1s 392-410 eV) and Copper (XPS: Cu 2p 910-970 eV, Auger: 560-580 eV) were obtained after cleaning surface with Ar-clusters (1000 atoms, 6000 eV, 1 mm raster size) for 60 s followed by data collection with a step size of 0.1 eV and a pass energy of 50 eV. All measurements were performed using Al-Kα X-rays with a spot size of 400 µm. Obtained spectra were evaluated using the Advantage software package v5.9929 provided by Thermo Fisher Scientific and Origin Pro v9.7.5.184.

Nuclear Magnetic Resonance spectra (NMR) were recorded on either a Bruker BioSpin AV III 600 (^{1}H-NMR: 600.25 Hz, ^{13}C-NMR: 150.93 Hz) or Bruker BioSpin AV neo 500 (^{1}H-NMR: 500.32 Hz, ^{13}C-NMR: 125.81 Hz) provided by the NMR Center, Faculty of Chemistry, University of Vienna. The residual solvent peaks were used as a reference. The resulting spectra were processed using TopSpin v4.1.1 and analyzed with MestReNova v14.1.2.

Mass spectrometry (MS) was performed on a Brucker amaZon speed ETD (reaction control/routine measurements) or Bruker maXis UHR-TOF (high resolution) spectrometer at Mass Spectrometry Centre, Faculty of Chemistry, University of Vienna.

UV-Vis spectroscopy was conducted on an Agilent Technologies G1103A using air-tight quartz cuvettes.

Transmission electron microscopy (TEM) was performed at the Electron Microscopy Facility at IST Austria using a Phillips CM2000 (200kV) TEM equipped with a Gaten Orius SC600 camera. Prior to imaging, samples suspended in toluene were drop casted onto carbon film on 200 mesh copper grids. The resulting pictures were processed with Gaten Micrograph software and analyzed with TVIPS EM Measure beta 0.85. The average particle diameter (d) and size distribution were determined by the analysis of obtained micrographs with ImageJ v1.53k.

Synthesis of NHC Complex 2

For the synthesis of the NHC-copper-complex **2** the procedure from Lu *et al.*[2] was adjusted. Therefore copper(I)oxide (0.19 mmol, 27 mg, 1 eq) was suspended in 1 mL of toluene and heated to 110 °C. 1,3-di(dodecyl)imidazole-1-ium bromide **1** (0.3 mmol, 100 mg, 1.6 eq) was added in one portion to the suspension. The reaction mixture was stirred for 24 h and filtered through silica resulting in a clear solution. The solvent was removed under reduced pressure resulting in a pale yellow, air-sensitive solid which was used without further purification. The product was obtained as off-white solid (100 mg, 61%).

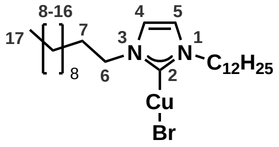

2
NHC-Cu(I)-Br

^{1}H-NMR (600 MHz, $CDCl_3$) δ = 6.88 (s, 2 H, $C^{4/5}H$), 4.10 (t, J = 7.3 Hz, 4 H, C^6H_2), 1.82 (t, 4H, J = 7.2 Hz, C^7H_2), 1.35-1.21 (m, 36 H, $C^{8\text{-}15}H_2$), 0.88 (t, J = 7.0 Hz, 3 H, $C^{16}H_3$) ppm.

^{13}C-NMR (600 MHz, $CDCl_3$) δ = 177.1 (C^2), 120.4 ($C^{4/5}$), 51.7 (C^6), 32.1 (C^7), 31.7-22.8 ($C^{8\text{-}15}$), 14.3 (C^{16}) ppm.

Synthesis of NHC@CuNPs

Complex **2** (0.09 mmol, 46 mg, 1 eq) was dissolved in 1 mL THF and heated to 50 °C. The reducing agent $NH_3{\cdot}BH_3$ (0.1 mmol, 3 mg, 1.1 eq) was added in one portion and the mixture stirred for 24 h. The resulting nanoparticles are collected by centrifugation under inert atmosphere and re-dispersed in toluene. For the separation of unbound ligand, reducing agent and further impurities, the surfactant was decanted after 15 min of centrifugation at 12100 x g. The procedure was repeated 5 times. Final **NHC@CuNPs** were obtained upon redispersion in toluene as dark red liquid.

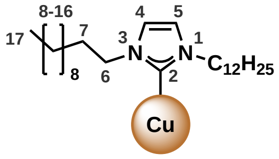

NHC@CuNP

^{13}C-NMR (600 MHz, $CDCl_3$) δ = 176.6 (C^2), 120.4 ($C^{4/5}$), 51.0 (C^6), 32.1 (C^7), 29.9-22.8 ($C^{8\text{-}15}$), 14.3 (C^{16}) ppm.

UV-Vis (toluene, λ_{max}) 584 nm.

TEM (n=100) 8.6 ± 1.6 nm.

Note: Both, complex **2** and **NHC@CuNPs** are air sensitive and can be only handled under inert atmosphere.

High Resolution XPS Spectra

All displayed spectra were deconvoluted using the Avantage software package (v5.9929/build 06752) provided by Thermo Fisher. Displayed baselines were generated by using the smart baseline feature of the software package. Raw data is displayed as gray symbol, generated envelope as red line and peak fits as colored areas. All high resolution XPS spectra were calibrated on the C-C contribution in C 1s spectra at 284.8 eV.

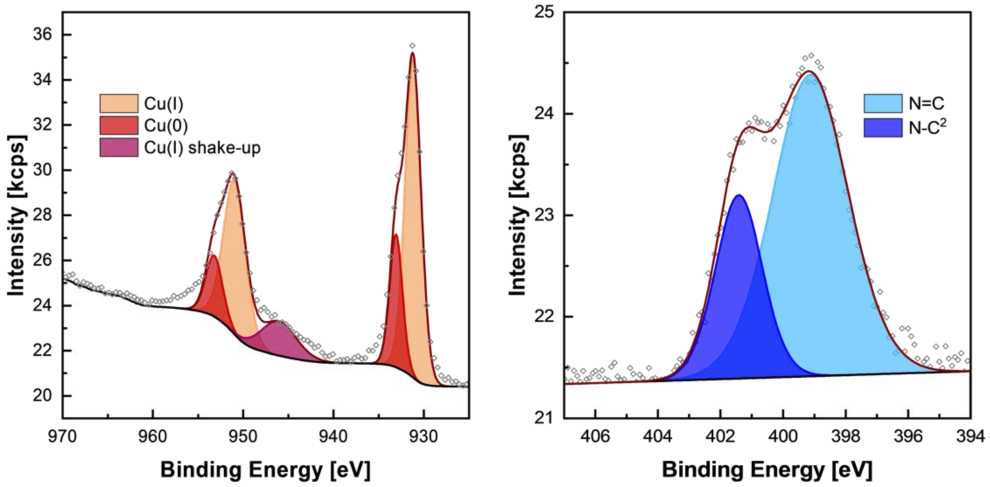

Figure S1 Cu 2p and N1 s spectra of **NHC@CuNP**s after air exposure.

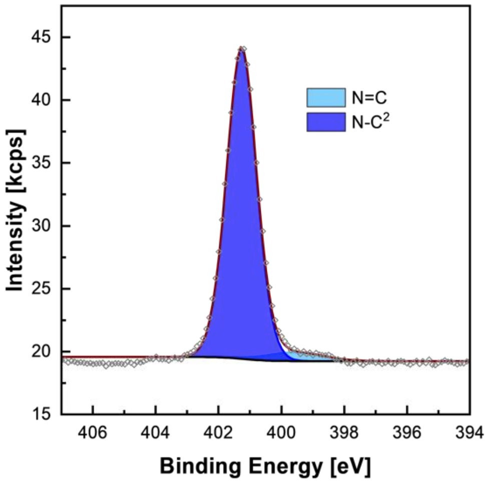

Figure S2 N 1s scan of imidazolium salt **1**.

XPS Survey Spectra of NHC@CuNPs

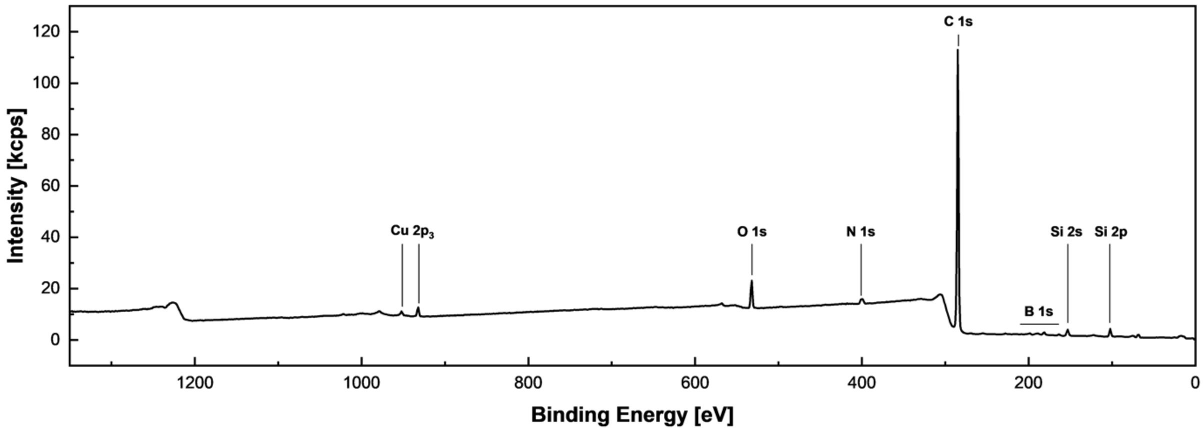

Figure S3 XPS Survey Spectrum of **NHC@CuNP**s.

UV-Vis Stability Study under Argon Atmosphere

Purified **NHC@CuNP**s in toluene were kept as stock solution under argon. The stability was assessed *via* UV-Vis spectroscopy by filling aliquots of the NP stock in air-tight cuvettes and recording spectra immediately as well as after 24 and 48 h. Samples were discarded after measurements.

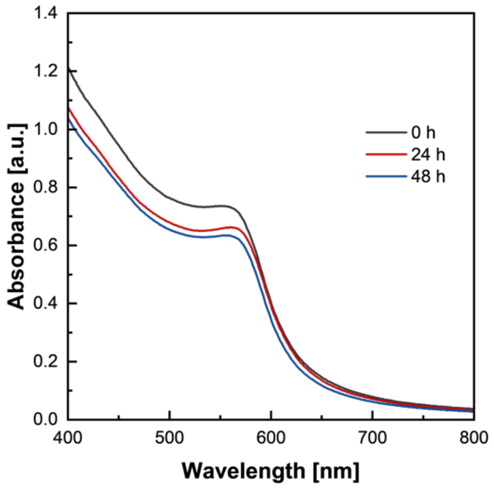

Figure S4 UV-Vis spectra of NHC@CuNPs stored under argon atmosphere.

TEM Micrograph of NHC@CuNPs

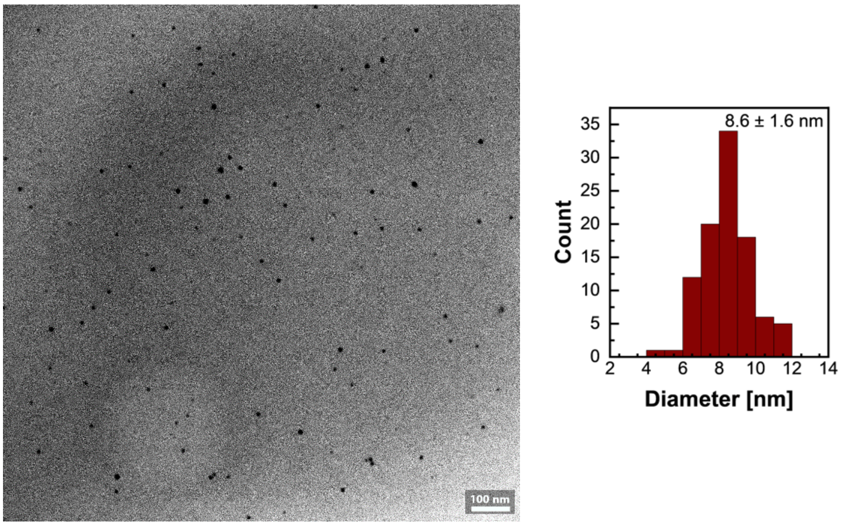

Figure S5 TEM micrograph of **NHC@CuNP**s and corresponding particle size histogram.

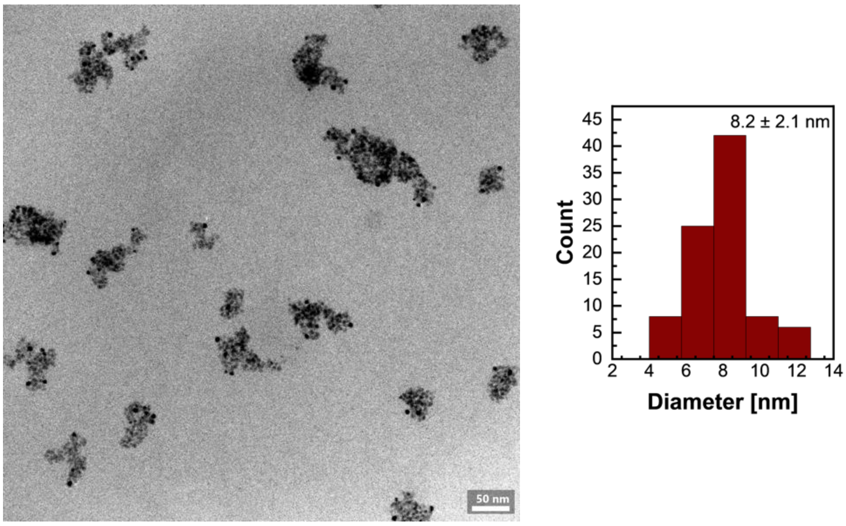

Figure S6 TEM micrograph of **NHC@CuNP**s after oxidation and corresponding particle size histogram.

^{13}C- and ^{1}H-NMR Spectra

Imidazolium salt **1**

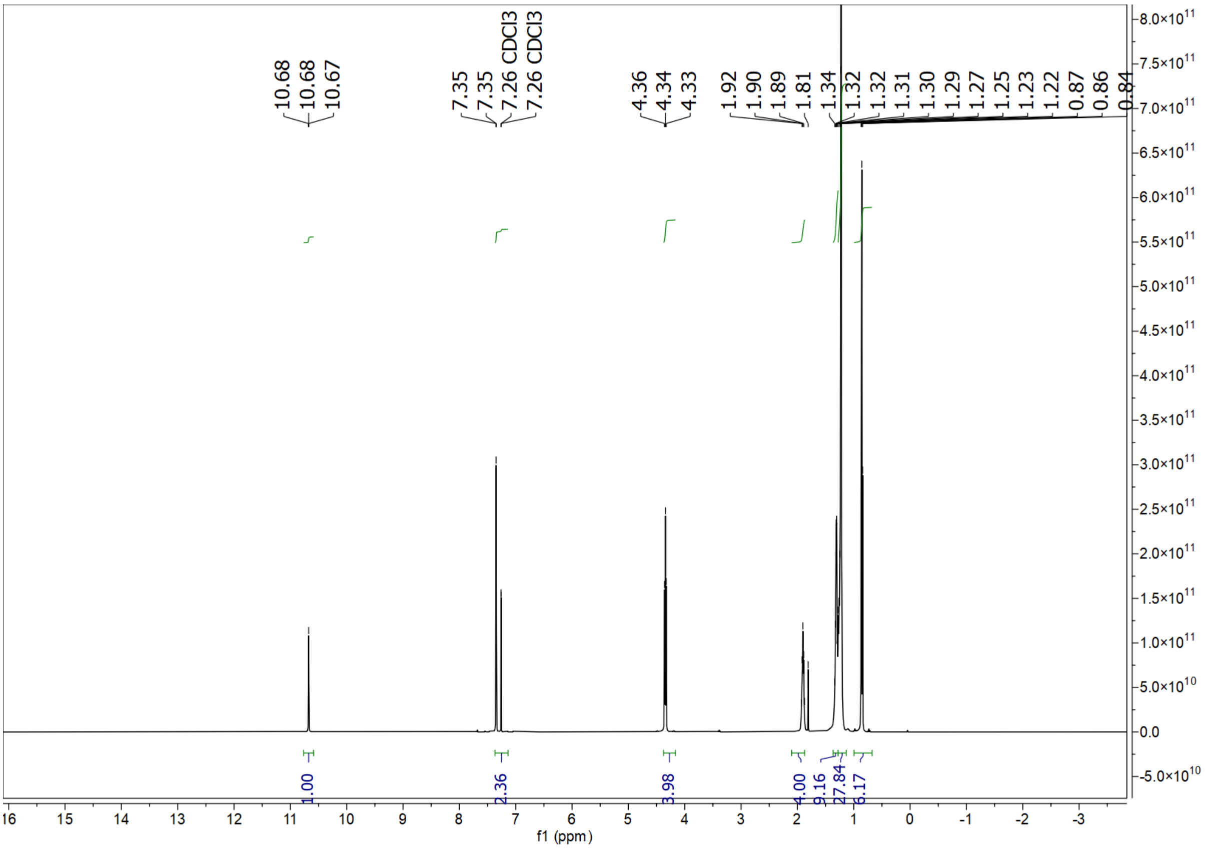

Figure S7 ^{1}H-NMR spectrum of 1,3-di(dodecyl)imidazole-1-ium bromide **1**.

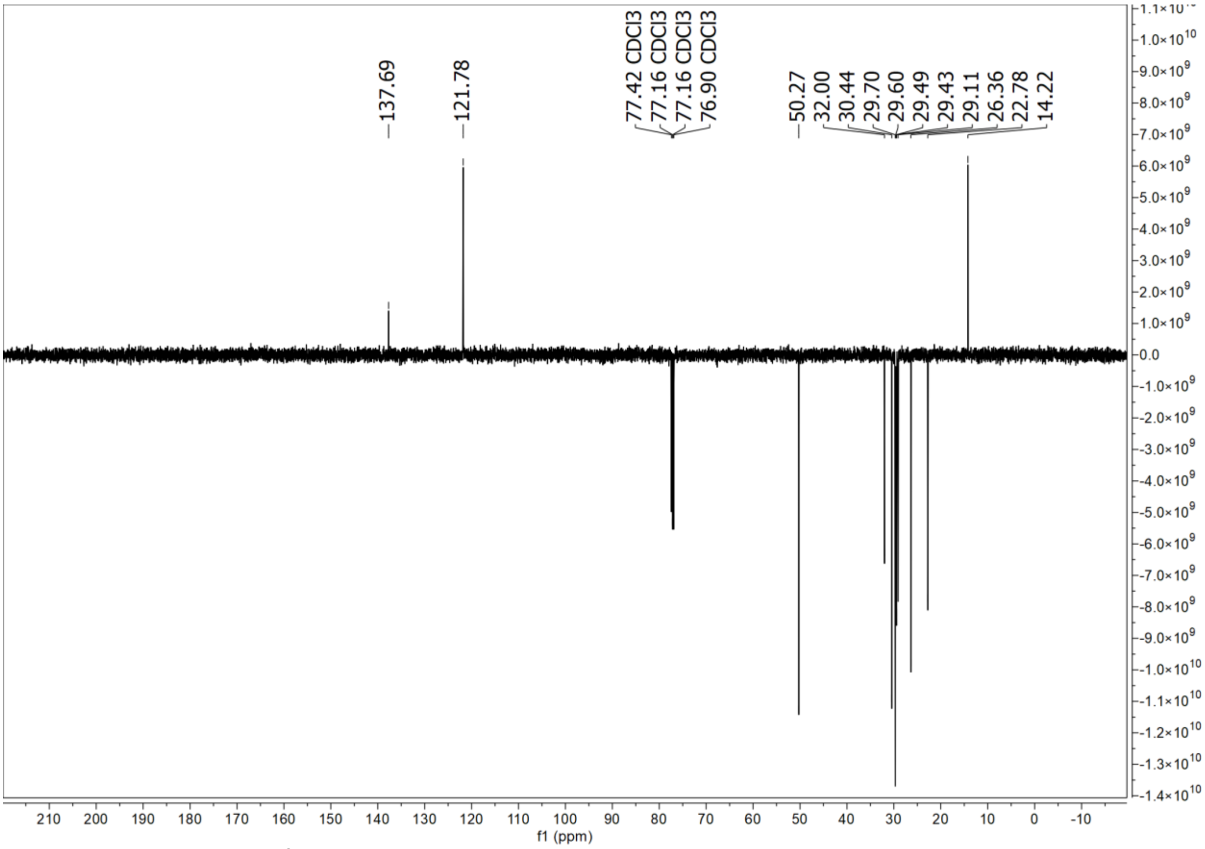

Figure S8 ^{13}C-NMR (JMOD) spectrum of 1,3-di(dodecyl)imidazole-1-ium bromide **1**.

NHC Complex **2**

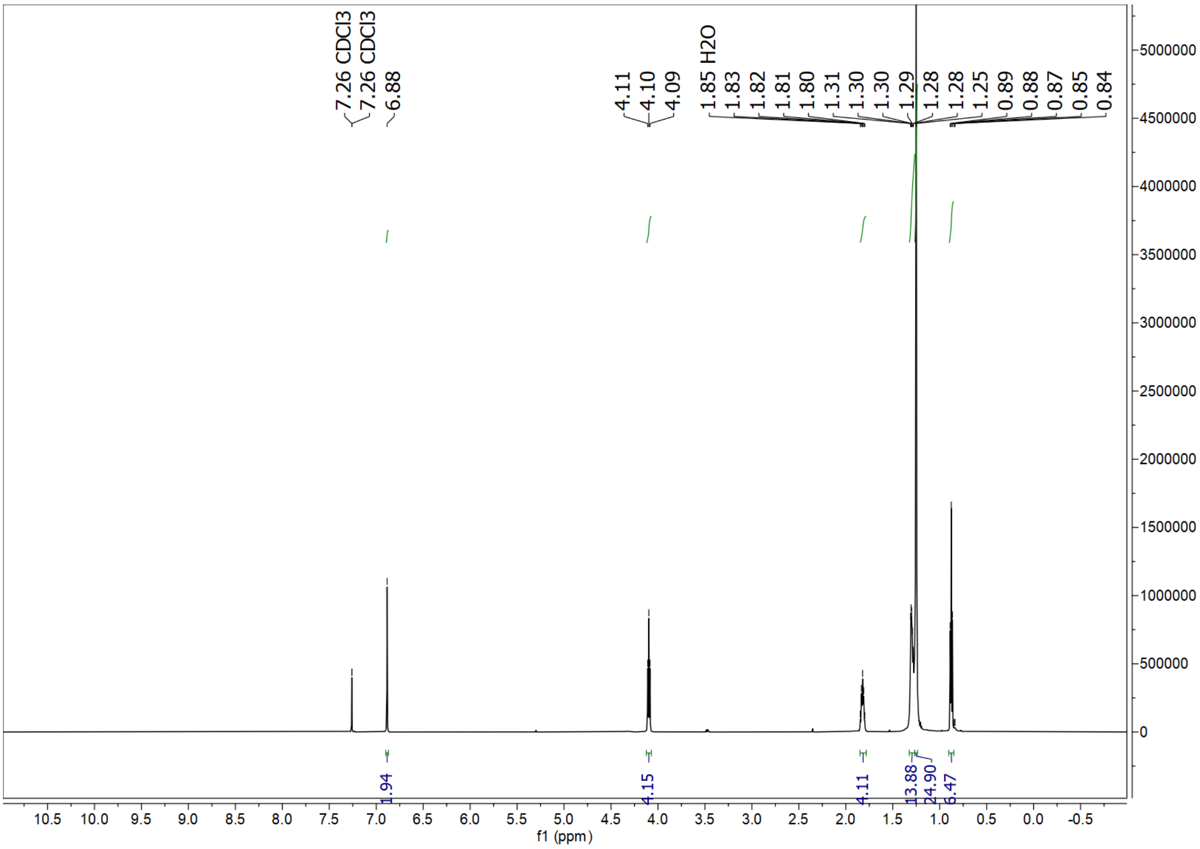

Figure S9 ^{1}H-NMR spectrum of 1,3-(diundecyl)-imidazole-2-yl)copper(II)bromide **2**.

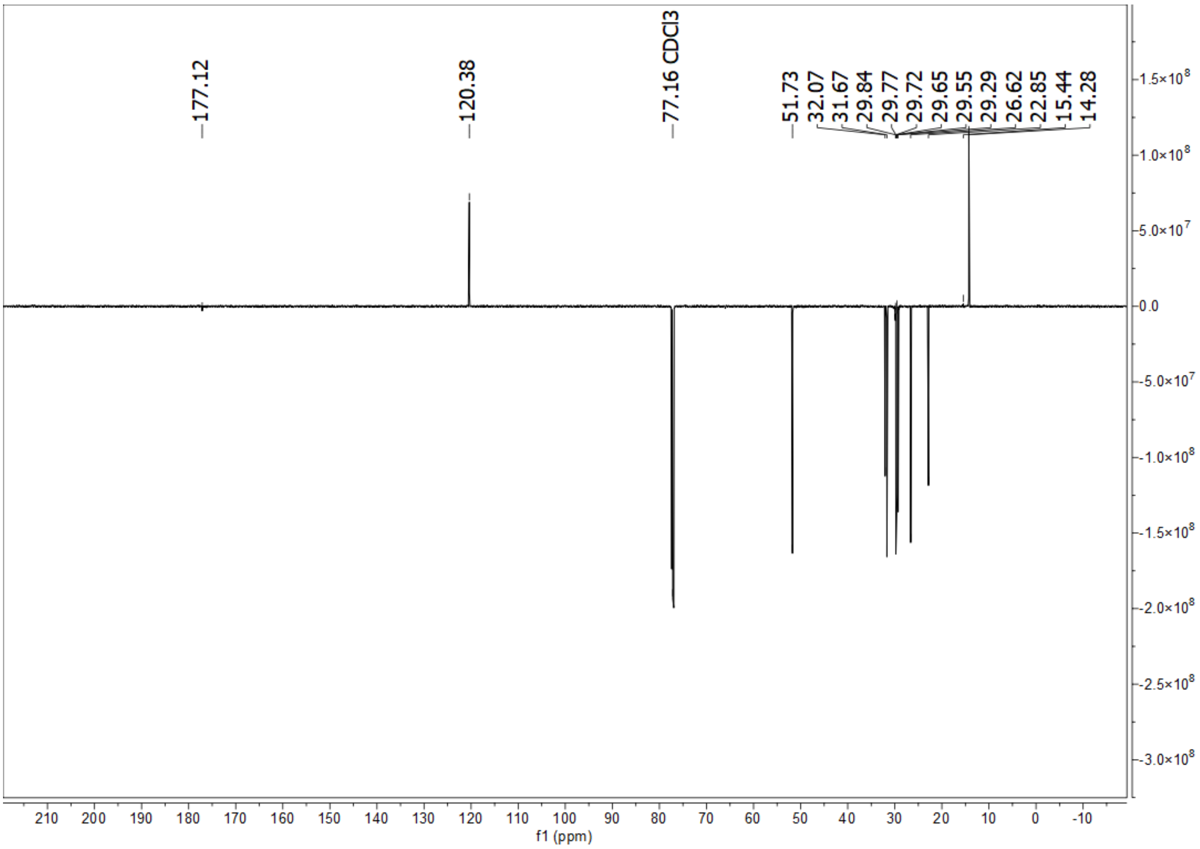

Figure S10 ^{13}C-NMR (JMOD) spectrum of 1,3-(diundecyl)-imidazole-2-yl)copper(II)bromide **2**.

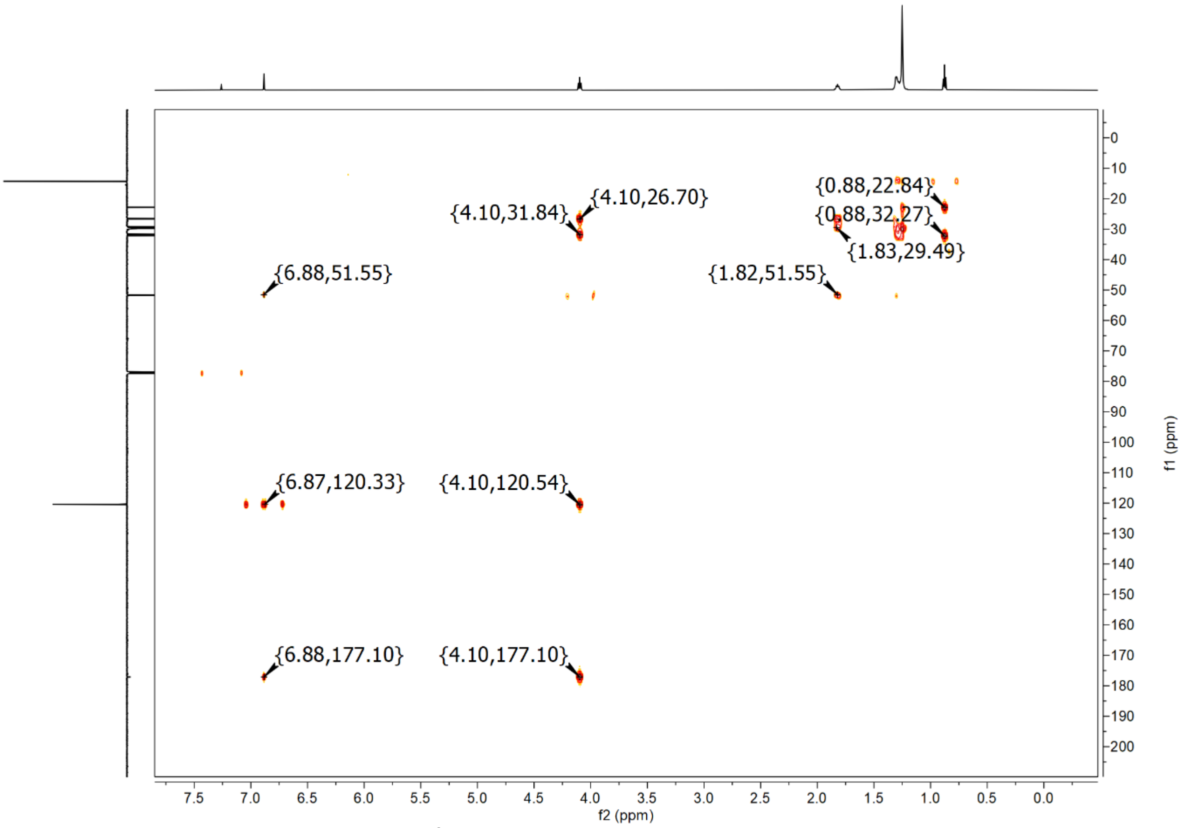

Figure S11 $^{1}H/^{13}C$-HMBC 2D spectrum of NHC complex **2**.

NHC@CuNPs

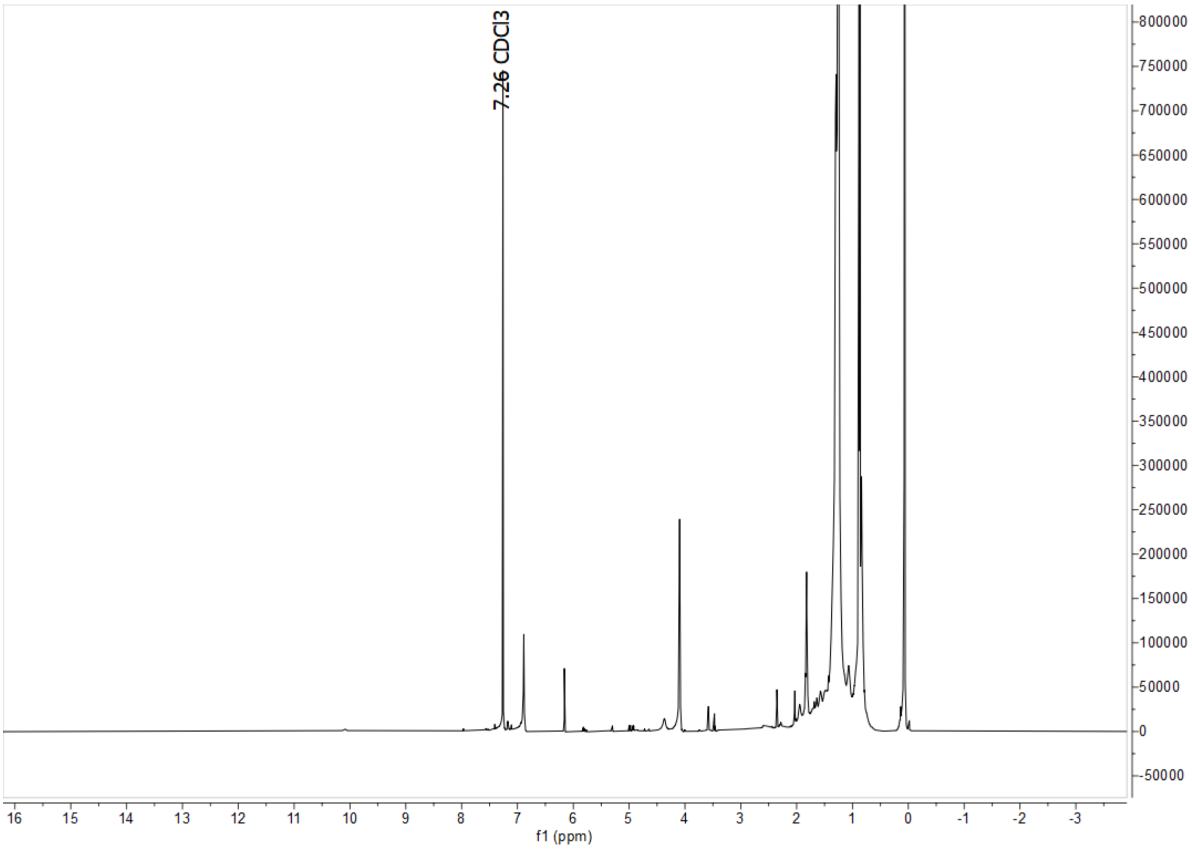

Figure S12 ^{1}H-NMR spectrum of **NHC@CuNP**s.

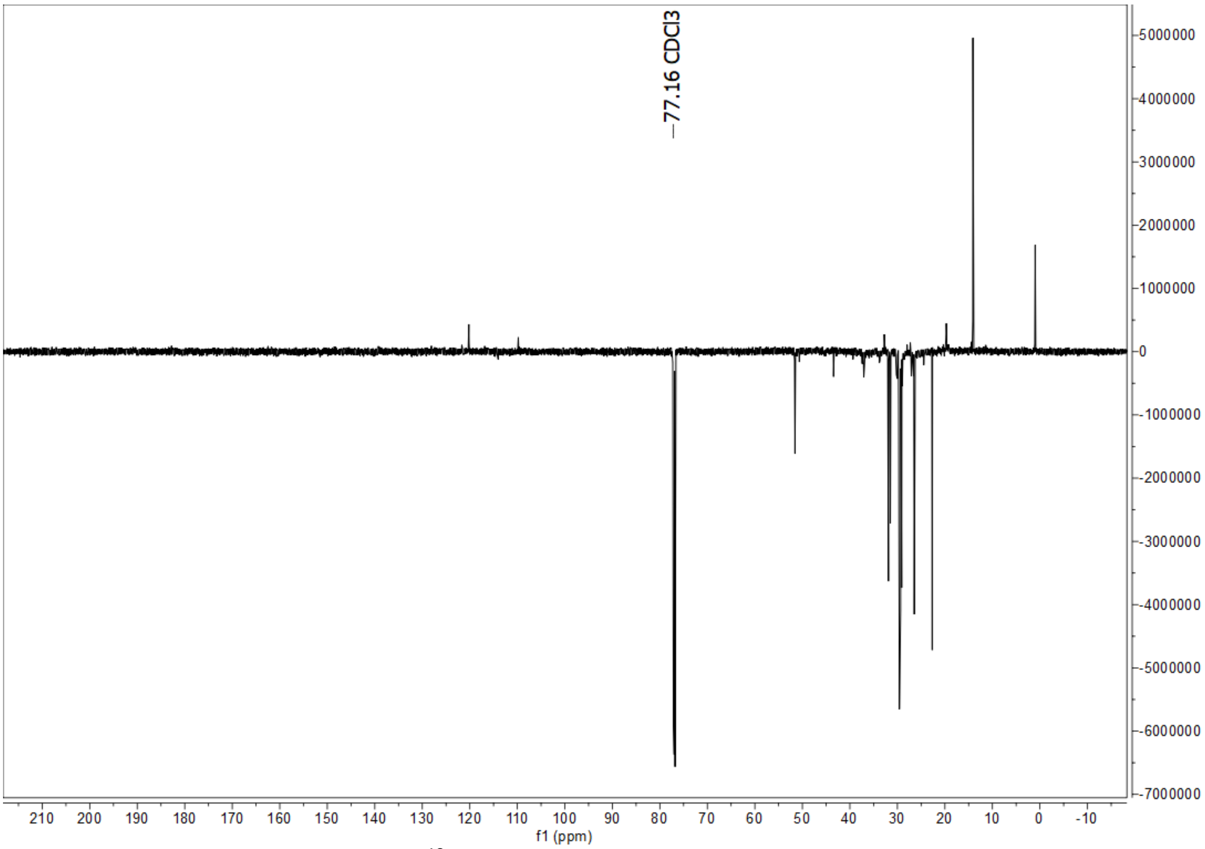

Figure S13 ^{13}C-NMR (JMOD) spectrum of **NHC@CuNP**s.

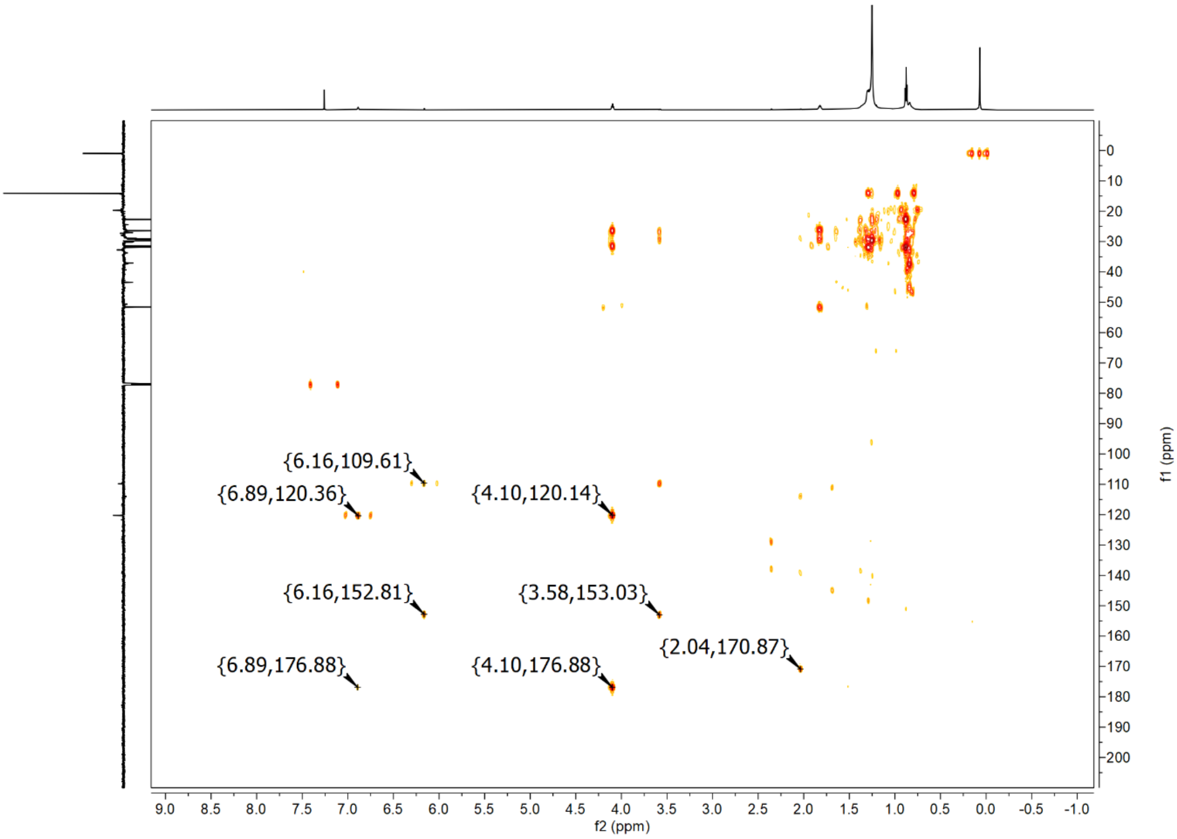

Figure S14 ^{1}H/^{13}C-HMBC 2D spectrum of **NHC@CuNPs**.

NHC@CuNPs after air exposure

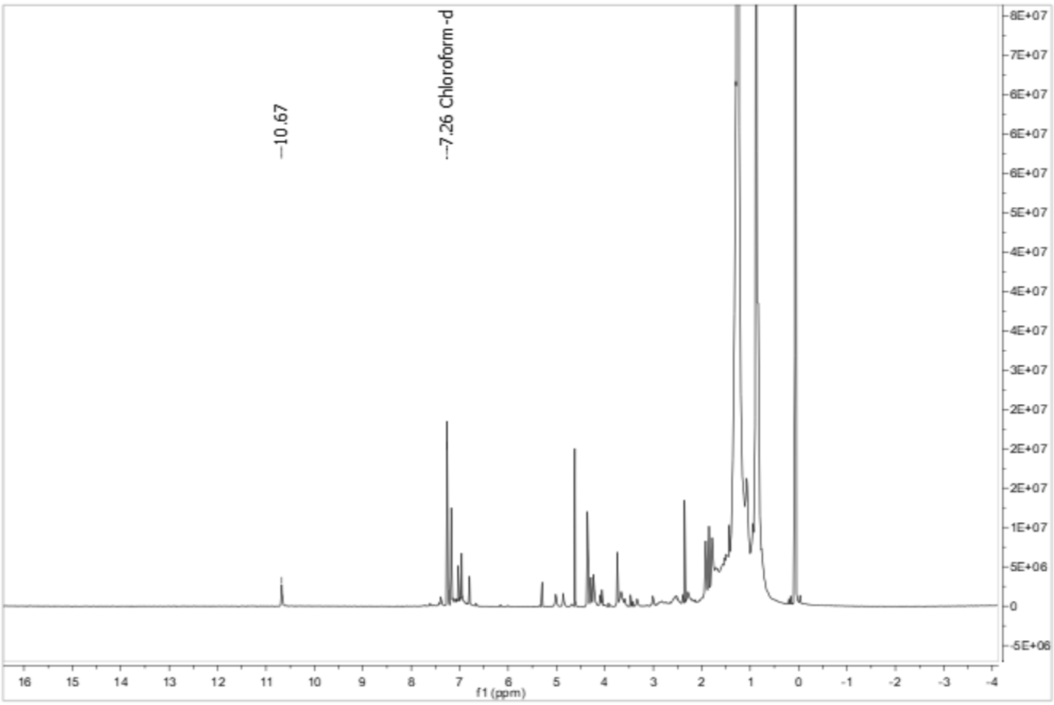

Figure S15 ^{1}H-NMR spectrum of **NHC@CuNP**s after oxidation.

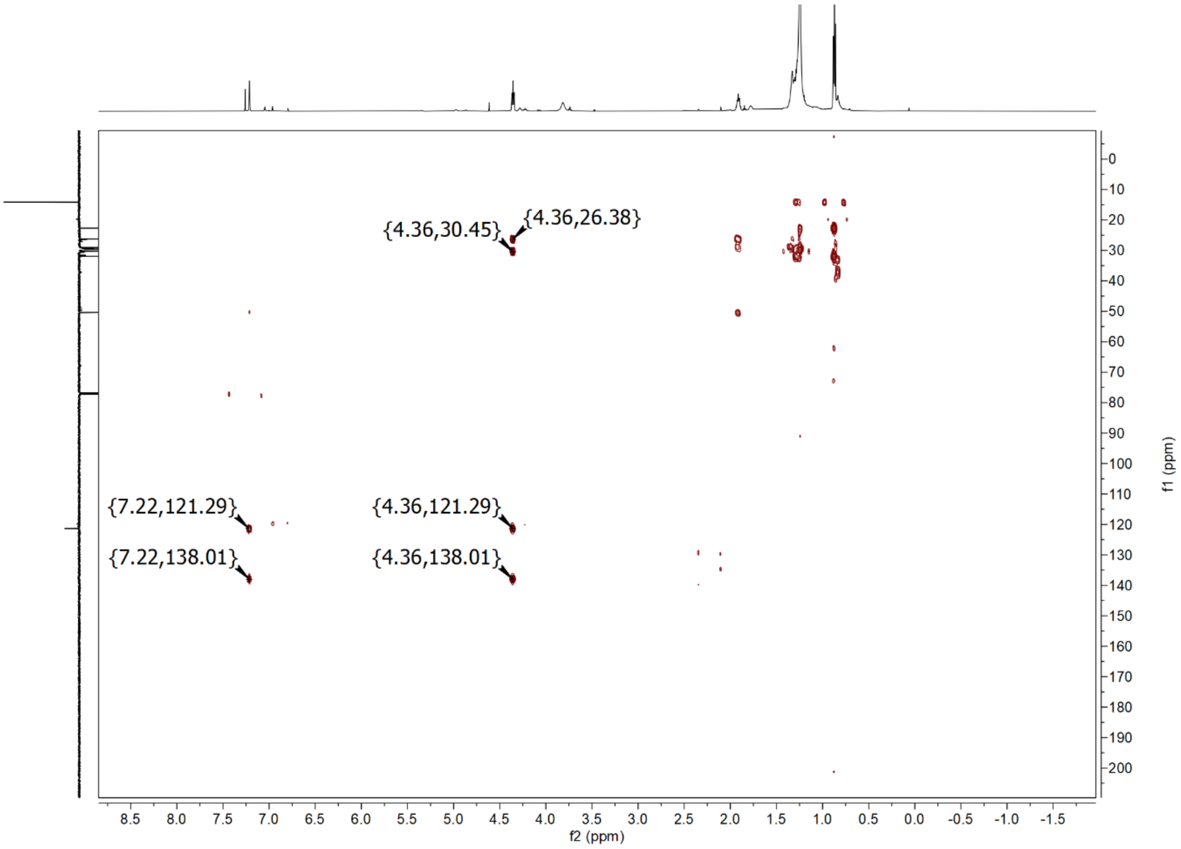

Figure S16 ^{1}H/^{13}C HMBC 2D spectrum of **NHC@CuNP** after oxidation.

High Resolution Mass Spectrometry of NHC@CuNP after Oxidation

Oxidized **NHC@CuNP**s were centrifuged and the supernatant was analyzed by HS-MS.

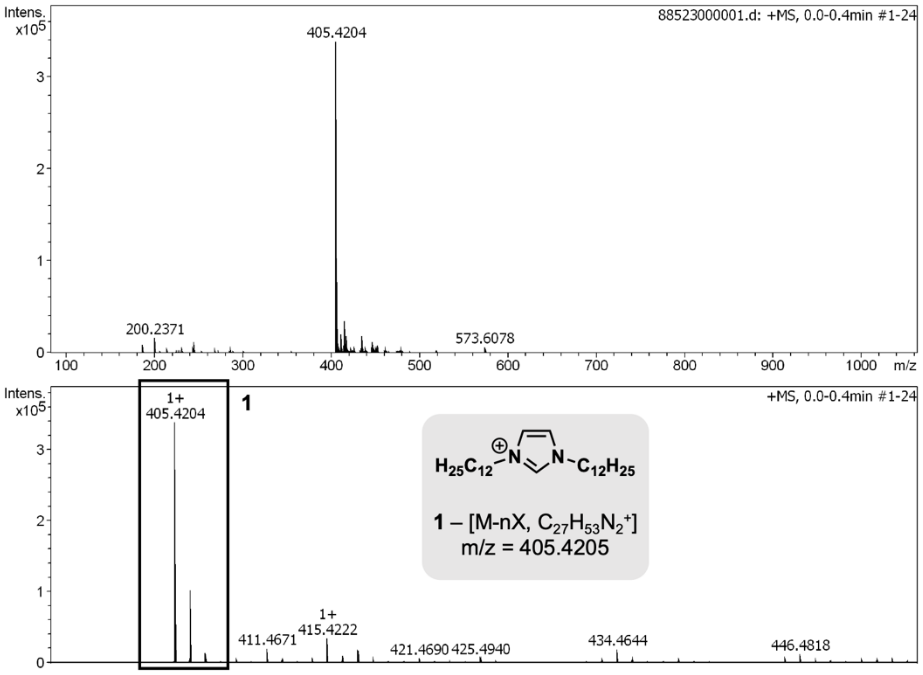

Figure S17 HS-MS spectra of NHC@CuNP supernatant after oxidation.

References

[1] K. Mebrouk, F. Camerel, O. Jeannin, B. Heinrich, B. Donnio, M. Fourmigue, *Inorg. Chem.* **2016**, *55*, 1296-1303.

[2] a) H. Lu, R. L. Brutchey, *Chem. Mater.* **2017**, *29*, 1396-1403; b) J. Chun, H. S. Lee, I. G. Jung, S. W. Lee, H. J. Kim, S. U. Son, *Organometallics* **2010**, *29*, 1518-1521.
